# Oral-Health-Related Self-Efficacy among the Elderly Population in Riyadh, Saudi Arabia

**DOI:** 10.3390/ijerph192315900

**Published:** 2022-11-29

**Authors:** Lamyia Anweigi, Alhanoof Aldegheishem, Ambreen Azam, Yara Alromaih, Fatima Alkeait, Lama Alhaimy, Ahmad Ahmeda, Shaza Bishti, Faleh Tamimi, Raidan Ba-Hattab

**Affiliations:** 1 College of Dental Medicine, QU Health, Qatar University, Doha 2713, Qatar; 2 Department of Clinical Dental Sciences, College of Dentistry, Princess Nourah Bint Abdulrahman University, Riyadh 11671, Saudi Arabia; 3 Dental Materials, College of Dentistry, Margalla Institute of Health Sciences, Rawalpindi 46000, Pakistan; 4 Ministry of Health, Hail 55422, Saudi Arabia; 5 Department of Basic Medical Sciences, College of Medicine, Ajman University, Ajman P.O. Box 346, United Arab Emirates; 6 Center of Medical and Bio-Allied Health Sciences Research, Ajman University, Ajman P.O. Box 346, United Arab Emirates; 7 Department of Prosthodontics and Biomaterials, Aachen University Hospital, 52074 Aachen, Germany

**Keywords:** dental health, elderly, oral health, self-efficacy, Saudi Arabia

## Abstract

Oral health self-efficacy is a fundamental determinant of behavioral changes among elderly patients. Objective: To assess the oral self-efficacy among the Saudi population aged 65 years old and above in Riyadh, Saudi Arabia. Methodology: This was a cross-sectional survey conducted on elderly individuals in Riyadh. An Arabic version of the Geriatric Self-Efficacy Scale for Oral Health (GSEOH) was administered to all participants. The dependent variables included oral function, oral hygiene habits, and dental visits. For the statistical analysis, two independent sample *t*-tests and a one-way ANOVA test were used. Significance was judged at a *p*-value less than 0.05. Results: Of 400 participants recruited, 53% were males. About 58% had retained teeth, and 72% had visited a dentist in the past 12 months. Overall, 31.6%, 34.64%, 22.65%, and 11.14% of the participants rated their oral health as good, fairly good, rather poor, and poor, respectively. Age (*p* < 0.001), educational level (*p* < 0.001), and working status (*p* < 0.001) were significantly associated with GSEOH scores. Other sociodemographic characteristics were not found to affect the GSEOH scores. Conclusions: The overall self-efficacy of oral health among Saudi elderly individuals is fairly good. Age, educational level, and occupational status are the main determinants of oral health self-efficacy scores.

## 1. Introduction

The Saudi Arabia population is rapidly aging [1]. The proportion of individuals aged 65 years or above is expected to expand progressively over the next couple of decades to make up approximately 18.4% of the total population of Saudi Arabia by 2050 [2]. With such a dramatic expected increase, there is an increased interest in studying Saudi older adults’ perceptions of aging [3,4]. This demographic transition should be considered in providing preventive and health care services including oral health services [5]. Oral health is an integral part of overall health and well-being [6]. Older adults experience more illness and poorer health compared to other age groups [5]. They present poor oral health manifested as high levels of dental caries, periodontal disease, tooth loss, xerostomia, and oral cancer [7,8,9]. The oral health of older adults can be improved, by providing access to care, health promotion, improvement in oral health knowledge, and self-managed disease-preventive measures [7,8,9]. All these factors are important for achieving, improving, and maintaining better oral health status and oral function as well as prevention of oral disease among older adults [4,5,6,8,9]. 

Perceived self-efficacy is defined as ‘the situation-specific confidence that individuals have about their personal ability to perform a behavior’, and it is an important factor influencing health behavior changes [5,6]. The relationship between the perception of self-efficacy and behavioral changes has been widely studied [10,11], based on the theory that health is determined by a certain interaction between behavioral, individual, and environmental factors [10,11]. The Wiedenfeld and Kiyak self-efficacy scale has commonly been used to measure the self-efficacy of oral health [4,12] and to explain patients’ oral health behavior in relation to the prevention of oral diseases such as periodontitis [13]. 

The Geriatric Self-Efficacy Scale for Oral Health (GSEOH- scale) was developed as an adaptation of the Self-Efficacy scale specifically designed to evaluate self-efficacy for oral health among older adults [6]. GSEOH scale focuses on three main important principles: oral function, oral hygiene habits, and dental visits [6]. It is considered a reliable and valid scale that was used with older adults by many studies [6,14,15]. 

The older population in Saudi Arabia holds positive views on aging [4], even though physical activity, financial resources, and daily responsibilities still raised considerable concerns [4]. 

The aim of this study was to assess the oral self-efficacy among the Saudi population aged 65 years old and above in Riyadh, Saudi Arabia, by using the GSEOH. The hypothesis of the study was that self-reported oral health status using GSEOH might be correlated with demographic, socioeconomic, and oral health factors in a population of elderly individuals aged 65 years and older living in Riyadh, Saudi Arabia.

## 2. Materials and Methods

### 2.1. Study Design and Population

A cross-sectional study was conducted in Riyadh, Saudi Arabia in 2017, among Saudi independent older people aged 65 years and above. Inclusion criteria included males and females aged 65 years or older living in Riyadh and functioning independently in the community.

### 2.2. Survey Structure

An Arabic version of the GSEOH developed by Yuki Ohara and Naomi Yoshida was used [6]. A section enquiring about sociodemographic information was added to the survey, and hard and soft copies were distributed to the participants. The dependent variable was the oral health self-efficacy measured using GSEOH, which consisted of 20 items divided into three main sections, i.e., oral function (box 1), oral hygiene habits (box 2), and dental visits (box 3). The response options for each item were evaluated on a four-point Likert scale, i.e., good (coded as 1), rather good (coded as 2), rather poor (coded as 3), and poor (coded as 4). Independent variables included sociodemographic data, age, and nationality, level of education, living arrangements, health insurance, working status, and economic status. In addition, three dental-related questions were also included in the survey. These include questions on whether the participants were dentate or not, preference of dental treatment (either ‘public hospitals’ or ‘private clinics’), and if participants had any recent dental visits within the past 12 months (a ‘yes’ or ‘no’ dichotomous variable).

### 2.3. Sample Size and Statistical Analysis

Sample size calculation determined that a sample size of 400 participants provides sufficient statistical power for our study. 

The sample size was calculated based on the study population’s age and gender. The estimated number of independent older people aged 65 years or more living in Saudi Arabia in 2017 was 165,776) according to population surveys. In order to calculate the minimum sample size required to be surveyed, the formula of Cochran was applied as follows:

(1) $$\overset{´}{n}=\frac{Z^{2}\times p\times \left(1-p\right)}{\varepsilon^{2}}$$

where *Z* is the *Z*-score based on the value of confidence, at a 95% confidence level (*Z* = 1.96); *p* represents the proportion of the population that has the attribute in question, which was considered *p* = 0.5 in this study; and ε is the level of marginal error, assumed to be 0.05.

Hence, the preceding figures were applied in the Cochran equation as follows.

(2) $$\overset{´}{n}=\frac{{\left(1.96\right)}^{2}\times 0.5\times \left(1-0.5\right)}{{\left(0.05\right)}^{2}}=384$$

Given that if there were 165,776 people within the targeted population (*n*), the modified Cochran equation was substituted as follows:

(3) $$n=\frac{\overset{´}{n}}{1+\frac{\left(\overset{´}{n}-1\right)}{N}}=\frac{384}{1+\frac{\left(383\right)}{165,776}}=\frac{384}{1+0.002310}$$

This calculation shows that at least 383 independent Saudi individuals aged 65 years or more from Riyadh were needed for our study.

Participants were selected by convenience sampling techniques. All data were fed into a computer and analyzed with the software SPSS software version 22.0. Qualitative data were expressed as frequency and percentages. The mean was used to express quantitative data. Two independent samples’ *t*-tests were used to compare mean scores of the GSEOH scale among participants within different age groups, different educational levels, and different economic states when comparing between two groups. A one-way ANOVA test was used to compare mean GSEOH scores based on their educational level and ages when a comparison between more than two groups was indicated. Significance was judged at a *p*-value less than 0.05.

### 2.4. Ethical Considerations

Ethical approval was obtained from the ethical committee of Princess Nourah Bint Abdulrahman University (Approval Nr. H-01-R-059). Informed consent was obtained from all patients to use their anonymous data for research purposes.

## 3. Results

### 3.1. Descriptive Analysis

#### 3.1.1. Sociodemographic Characteristics

Of the 400 participants, recruits for our study that fulfilled our inclusion and exclusion criteria, half of them (53%) were males, more than two-thirds (67%) were between 65 and 70 years old, and at least one-third of them (36.5%) had primary school education (Table 1). The vast majority of the sample (85%) lived with others, and only 35% were covered by health insurance. Approximately one-third of the participants had a low income (i.e., less than 3000 Saudi Arabia Riyal (SAR)), and only 28.5% were employed. More than half of the sample preferred dental treatment in a public hospital (58.5%), whereas receiving treatment in a private clinic was only preferred by 41.5% of participants. About 42% were completely edentulous, participants had teeth at the time of study recruitment, and approximately three-fourths (72%) had visited a dentist during the 12 months prior to the survey (Table 1).

#### 3.1.2. Geriatric Self-Efficacy Scale for Oral Health Responses

Table 2 details the distribution of participants according to their self-perception on the three sections of the GSEOH scale. About half of the participants reported good or fairly good oral functions, and about two-thirds reported good or fairly good oral hygiene habits. The participants’ responses about dental visits were positive towards being keen on visiting dentists regularly. In general, 31.6%, 34.64%, 22.65%, and 11.14% of the participants rated their overall oral health as good, fairly good, rather poor, and poor, respectively. Most participants rated their oral health as good or rather good (Table 2). 

### 3.2. Impact of Socio-Economic Factors on Participants’ Oral Health

Two-independent samples’ student *t*-tests were used to compare the mean GSEOH scores among different genders, working states, living arrangements, dental visits in the past 12 months, dental treatments preference, presence of teeth, and insurance coverage. As depicted in Table 3, the oral health mean scores were not significantly different among different genders (*p* = 0.545), living arrangements (*p* = 0.742), insurance coverage (*p* = 0.595), or dental treatment preferences (*p* = 0.657). On the other hand, the oral health scores were significantly different among participants as a function of working status, frequency of dental visits during the past 12 months, and presence of teeth. Mean GSEOH scores were significantly higher among employed participants (3.106) than those who were not working (2.771) (*p* < 0.001). The scores were also significantly higher among patients who had visited a dentist in the past 12 months (2.978 versus 2.575, *p* < 0.001) and among patients with teeth (2.976 versus 2.714, *p* < 0.001).

The one-way ANOVA test was used to compare the mean GSEOH scores among different age groups, educational levels, and economic statuses (Table 4). Multiple comparisons were performed to identify the differences between the studied variables using Post Hoc tests such as Tukey HSD tests. The oral health measured by GSEOH was not affected by economic status (*p* = 0.342). However, the mean GSEOH scores were significantly different among participants of different age groups and those with different educational levels (*p* < 0.001).

Table 5 demonstrates that non-educated participants had significantly lower GSEOH scores than participants with primary school (*p* = 0.007), secondary school (*p* = 0.003), and diploma levels of education (*p* < 0.001). With regard to age, participants aged 65 to 70 years had lower GSEOH scores than participants above 75 years (*p* < 0.001). 

The multiple regression analysis showed that independent variables, namely presence of teeth, medical insurance, working and economic status, dental visits (within the last 12 months), living arrangement, age, gender, and educational status, explain 20.7% of the variability in the GSEOH scores variable. An ANOVA test was used to find out if the overall regression model is a good fit for the collected data. Table 6 revealed that the independent variables significantly predicted the dependent variable (GSEOH scores) (*p* < 0.001).

Unstandardized coefficients showed that the independent variables (age, educational level, working status, dental visits, and presence of teeth) significantly (*p ≤* 0.05) predicted GSEOH scores. Coefficients of the dependent and independent variables show significant association between GSEOH scores, and age, educational level, working status, dental visits, and presence of teeth (Table 7).

For example, the standardized coefficient for age variable is (*B*_1_ = −4.345) indicated that for each one-year increase in age, there was a decrease in GSEOH score of 4.345. On the other hand, the standardized coefficient for the educational level variable is (*B*_2_ = 1.598), which indicates that for each one-level increase in educational level, there was an increase in GSEOH score of 1.598.

Therefore, the final model for predicting GSEOH score based on background variables included in this study is

(4) $$\begin{array}{ll} GSEOH_{score}= & 83.438-\left(4.345\times Age\right)+\left(1.598\times Educational\;level\right)\\ & -\left(4.152\times Working\;status\right)-\left(4.642\times dental\;visits\right)\\ & -\left(4.278\times Presence\;of\;teeth\right) \end{array}$$

## 4. Discussion

The increasing elderly population in Saudi Arabia will result in a growing need for dental care services [2]. To the best of our knowledge, this study is the first to explore oral health knowledge, attitudes, and self-efficacy among older adults living in Riyadh, Saudi Arabia. Our study targeted the independent elderly; as a result, the elderly population of those aged 60 and above is projected to increase from 3% in 2010 to 9.5% and 18.4% in 2035 and 2050, respectively. Dental caries has been reported to be the most significant health problem facing the Saudi Arabian elderly population [9]. With such a high caries rate, urgent interventions are needed to encourage people to adopt preventive therapy and improve their knowledge, attitude, and self-efficiency. To adopt such measures, evaluation of the current oral health knowledge and attitudes is a fundamental initial step; this was the aim of this study. The main findings of this research were that the overall self-reported oral health was rated as fairly good among the participants, and the main determinants for oral health scores were age and educational levels. Oral health was not affected by sociodemographic characteristics. Hence, we could reject the hypothesis that there is no statistically significant difference among the elderly Saudi population aged 65 years or more living in Riyadh in terms of self-reported oral health using GSEOH. However, these findings must be interpreted with caution as 42% of all participants were edentulous. Moreover, the actual oral health condition of the participants remains unknown, as no data from clinical investigations were obtained in this study.

More than one-fourth of the participants recruited for this study had not visited the dentist during the past year. Similar findings have been previously reported in prior research in Saudi Arabia and were attributed to economic difficulties and limited access to health services [3,4,16,17,18,19,20]. Lack of supposed needs among the elderly population of Saudi Arabia was the most reported barrier to dental services. This figure of low access and utilization of dental services among the elderly population of Saudi Arabia might be reflected in their dental care [21]. Lack of insurance coverage was another significant cause of limited visits to the dentist. Shortage of money and lack of insurance were the most common factors that correlated negatively with access and utilization of dental services in other studies [22,23,24]. Official health policymakers might use these figures to establish a mechanism to increase access and enhance the utilization of healthcare services among the elderly [21]. In 2015, the WHO published the World Report on Ageing and Health, which established a framework for action to foster healthy aging [8]. The policies are highly relevant to the improvement in oral health. Transformation of oral health systems away from a disease-based curative model and towards disease prevention, as well as the provision of older-person-centered integrated care, are required. Moreover, wide-ranging public health action on aging is urgently needed [8]. 

In the present study, the mean GSEOH scores reflecting their evaluations towards oral health were significantly associated with their dental visits in the 12 months before participation in the study. This finding agrees with previous studies that reported that dental care cost and lack of awareness regarding services provided and the location of facilities had been significant barriers to the utilization of dental services among older adults [25,26]. In addition, oral health literacy, lack of a perceived need for care, disability, and dental fear were all reported to be essential factors influencing dental visits by the elderly population [27,28]. The most common barriers to dental services in Saudi Arabia were the lack of perceived need, no dental insurance, unaffordable price, transportation, and fear of dental treatment [21]. Additionally, the traditional role of an extended family is helpful for older persons to receive what they need from the service [4,16]. The current changes in family caregiving trends have created a lesser availability of potential caregivers, which had a potentially negative impact on dental care [4,16]. 

The number of retained teeth in older people serves as a measure of oral health. In this study, more than half of the participants above 65 years had retained teeth, which is similar to the proportion reported in Germany and Denmark [29,30]. Increasing the number of older adults with retained teeth would result in the continuous improvement in dental care services. Therefore, as more adults keep their teeth into advanced old age, the risk of contracting dental disease increases. Consequently, the need for dental care services for older people also increases [31]. The current study showed that participants’ oral health is associated with the presence of teeth. This agrees with other reports in the literature that noted a significant correlation between the reductions in the number of retained natural teeth and poor oral health [32,33,34,35,36,37]. However, having fewer teeth did not affect the quality of life (QoL) [37,38,39]. 

Age was a significant determinant of the oral health score in this study. In the literature, it has been reported that oral-health-related QoL (OHRQoL) decreases with age, and it was related to social class [40,41]. The impact of socioeconomic status on OHRQoL was conflicting in different studies and different populations [42,43]. Additionally, older people may ascribe a lower priority to oral health in comparison to general health and thus report less impact on their oral health than public health on QoL [44]. In our study, the economic state did not correlate significantly with oral health scores. 

Educational level was another significant determinant of oral health scores in our study. Health literacy skills are critical in maintaining the quality of life for the elderly population [45]. It was also shown to be an indispensable contributor to both general and oral health [46]. As individuals age, health literacy becomes a valuable tool to help take or administer medications appropriately [47]. The improvement in health literacy skills can be carried out through adult education, seminars, self-study, internet use, library use, daily reading, and engagement with social networks [45]. Moreover, reading books, magazines, and newspapers at home was found to have a more substantial effect on one’s health literacy than educational attainment [48]. These practices can be maintained through life and increase health knowledge relatively inexpensively [45]. The importance of lifelong learning is well-established in the literature, and it is receiving increased attention [37,38,39]. Developing an understanding of oral health knowledge, attitudes, and beliefs among older adults is the first step towards designing health promotion programs that guide policies and programs [42,49]. Additionally, self-efficacy expectations are positively and significantly associated with initiating and maintaining healthy behaviours [43,44,50,51]. Lifestyle modifications have been successfully implemented even in the very old, provided that comorbidities are not overwhelming [40,41]. Oral health education and prevention efforts must be tailored to specific subgroups of older adults. Generic health promotion efforts in the older population, without being specifically designed to meet the needs of subgroups within this population, will not achieve the desired outcomes [45]. In contrast, individualized and peer-led interventions have been demonstrated to be useful educational techniques to further identify and directly address misconceptions and to promote better attitudes [13,14,18,52]. 

The main limitation of this study was that no objective clinical findings were obtained. The presence of such information will strengthen the findings of this study by assessing if the self-reported measures are comparable with the clinical status of the participants.

Another limitation is that the participants recruited were older adults living in the capital city with higher economic status and better levels of healthcare services than other regions of the country, making the sample non-representative for the country of Saudi Arabia. The findings warrant further studies, including additional cities, to provide a reliable and holistic picture of oral health-related self-efficacy for older people in Saudi Arabia.

## 5. Conclusions

Self-reported oral health perception was fairly good among elderly individuals living in Riyadh, Saudi Arabia. Age and educational level, working status, dental visits, and the presence of teeth were the main determinants of oral health perception. However, clinical evaluation is necessary for making robust conclusions on the oral hygiene habits and oral health in this particular population. 

## Figures and Tables

**Table 1:** The distribution of the sample according to demographical and economic factors such as age, gender, educational level, and working status.

Variable		Frequency	Percentage (%)
Age	65–70 y	269	67
	70–75 y	68	17
	Above 75 y	63	16
Gender	Male	213	53
	Female	187	47
Education	No education	98	24.5
	Primary school	146	36.5
	Secondary school	76	19
	Diploma	66	16.5
	Higher education	14	3.5
Living	Alone	59	15
	With others	341	85
Insurance	Yes	140	35
	No	260	65
Dental treatment	Public hospitals	234	58.5
	Private clinics	166	41.5
Economic status	Less than 3000 SAR	128	32
	3000–6000 SAR	114	29
	6000–10,000 SAR	53	13
	10000–15,000 SAR	57	14
	More than 15,000 SAR	48	12
Working status	Yes	114	28.5
	No	286	71.5
Dental visits (last 12 months or less)	Yes	289	72
	No	111	28
Presence of some teeth	Yes	233	58
	No	167	42
	No		

y: Years; SAR: Saudi Arabia Riyal.

**Table 2:** Distribution of participants according to self-perception using GSEOH scale.

Question	Frequency (Percentage)			
	Good (1)	Rather Good (2)	Rather Poor (3)	Poor (4)
Oral functioning				
I can talk smoothly	211 (53)	117 (29)	51 (13)	21 (5)
I can recover quickly after feeling bad about my mouth or teeth	115 (29)	161 (40)	80 (20)	44 (11)
I can enjoy daily life even with oral problems	155 (39)	150 (37)	67 (17)	28 (7)
I can talk with others without worrying about my mouth	183 (46)	119 (30)	69 (17)	29 (7)
I can speak easily even with a dry mouth	137 (34)	147 (37)	79 (20)	37 (9)
I can swallow easily even without a drink or soup	157 (39)	133 (33)	71 (18)	39 (10)
I am very confident about my mouth	120 (30)	147 (37)	94 (23)	39 (10)
I can enjoy eating	161 (40)	145 (36)	61 (15)	33 (9)
I can chew anything without a problem	155 (39)	144 (36)	67 (17)	34 (8)
Oral hygiene habits				
I keep my mouth clean	171 (43)	149 (37)	47 (12)	33 (8)
I practice oral care even when I’m busy	128 (32)	133 (33)	100 (25)	39 (10)
I make fine motions with a toothbrush	118 (29)	143 (36)	82 (21)	57 (14)
I use special techniques to brush my teeth	66 (16.5)	124 (31)	142 (35.5)	68 (17)
I can check the cleanliness of my mouth	124 (31)	154 (38.5)	70 (17.5)	52 (13)
I listen to and follow necessary advice for oral health	105 (26)	149 (37)	97 (24)	49 (13)
I rinse my mouth after each meal	144 (36)	165 (41)	54 (14)	37 (9)
I can observe the cleanliness of my tongue	105 (26)	149 (37)	100 (25)	46 (12)
Dental visits				
I will continue visiting the clinic periodically even to prevent a recurrence	69 (17)	122 (30.5)	147 (37)	62 (15.5)
I go for routine check-ups for oral health	58 (14.5)	118 (29.5)	158 (39.5)	66 (16.5)
I go for routine check-ups even when busy	44 (11)	103 (26)	176 (44)	77 (19)
Overall oral health				
	126 (31.6)	139 (34.64)	91 (22.65)	44 (11.14)

**Table 3:** Comparison of the mean GSEOH scores among different genders, working states, living arrangements, dental visits in the past 12 months, dental treatments preference, presence of teeth, and insurance coverage.

Variable	Mean	Mean Difference	Levene’s Test for Equality of Variances		T-Test for Equality of Means	
			F	Significance	T	Significance
Gender						
Male	2.886	0.042	1.623	0.203	0.606	0.545
Female	2.844					
Living arrangements						
Alone	2.839	−0.032	0.004	0.949	−0.330	0.742
With others	2.871					
Insurance						
Yes	2.892	0.039	0.191	0.662	0.532	0.595
No	2.853					
Dental treatment						
Public hospitals	2.857	−0.031	1.599	0.207	−0.444	0.657
Private clinics	2.889					
Working status						
Yes	3.106	0.334	11.603	0.001	4.421	0.000 *
No	2.771					
Dental visits (last 12 months)						
Yes	2.978	0.402	37.19	0.000	5.333	0.000 *
No	2.575					
Presence of teeth						
Yes	2.976	0.262	9.071	0.003	3.756	0.000 *
No	2.714					

* Significant difference.

**Table 4:** Comparison of the mean GSEOH scores among different age groups, educational levels, and economic status using the one-way ANOVA test.

Variable	Source of Variation	Levene’s Test	Difference	Mean Square	F	Significance
Economic status	Between groups	0.141	5	0.554	1.134	0.342
	Within groups		394	0.488		
	Total		399			
Educational level	Between groups	0.053	4	2.822	6.061	0.000 *
	Within groups		395	0.466		
	Total		399			
Age	Between groups	0.000	2	12.650	29.562	0.000 *
	Within groups		397	0.428		
	Total		399			

* Significant difference.

**Table 5:** Post Hoc Tukey HSD analysis for GSEOH scores according to educational levels and age factors.

Post Hoc Tukey HSD	Variable	Level i	Level j	Mean difference	Significance
	Educational level	No education	Primary school	−0.303	0.007 *
		No education	Secondary school	−0.374	0.003 *
		No education	Diploma	−0.470	0.000 *
	Age	65–70 years	Above 75	−0.700	0.000 *
		70–75 years	Above 75	−0.491	0.000 *

* Significant difference.

**Table 6:** ANOVA test for the relationship between GSEOH scores and the study independent variables.

Model	Sum of Squares	df	Mean Square	F	Significance
Regression	16141.093	10	1614.109	10.139	0.000 *
Residual	61930.017	389	159.203		
Total	78071.110	399			

* Significant difference (presence of teeth, insurance, working status, economic status, dental visits (last 12 months or less), living arrangement, gender, preference of dental treatment, age, educational level).

**Table 7:** Multiple regression analysis for the association between GSEOH scores and the study’s independent variable.

Model	Unstandardized Coefficients		Standardized Coefficients	t	Significance
	B	Std Error	Beta		
Age	−4.345	0.931	−0.234	−4.667	0.000 *
Gender	−0.302	1.368	−0.011	−0.221	0.825
Education level	1.598	0.681	0.129	2.346	0.019 *
Living arrangement.	−0.208	1.879	−0.005	−0.111	0.912
Insurance	−0.183	1.350	−0.006	−0.135	0.892
Preference of dental treatment	−1.482	1.300	−0.055	−1.140	0.255
Economic status	−0.422	0.437	−0.050	−0.966	0.334
Working status	−4.152	1.497	−0.134	−2.775	0.006 *
Dental visits (last 12 months)	−4.642	1.505	−0.149	−3.084	0.002 *
Presence of teeth	−4.278	1.317	−0.151	−3.249	0.001 *

* Significant differences.

## Data Availability

The data presented in this study are available on request from the corresponding author.
